# A Chromosome-Scale Reference Assembly of a Tibetan Loach, *Triplophysa siluroides*

**DOI:** 10.3389/fgene.2019.00991

**Published:** 2019-10-16

**Authors:** Liandong Yang, Ying Wang, Tai Wang, Shengchang Duan, Yang Dong, Yanping Zhang, Shunping He

**Affiliations:** ^1^The Key Laboratory of Aquatic Biodiversity and Conservation of Chinese Academy of Sciences, Institute of Hydrobiology, Chinese Academy of Sciences, Wuhan, China; ^2^School of Life Sciences, Jianghan University, Wuhan, China; ^3^Gansu Key Laboratory of Cold Water Fishes Germplasm Resources and Genetics Breeding, Gansu Fishers Research Institute, Lanzhou, China; ^4^Nowbio Biotechnology Company, Kunming, China; ^5^Center for Excellence in Animal Evolution and Genetics, Chinese Academy of Sciences, Kunming, China

**Keywords:** *Triplophysa siluroides*, PacBio sequencing, genome assembly, evolution, adaptation

## Abstract

Cobitoidea is one of the two superfamilies in Cypriniformes; however, few genomes have been sequenced for Cobitoidea fishes. Here, we obtained a total of 252.90 Gb of short Illumina reads and 31.60 Gb of long PacBio Sequel reads, representing approximate genome coverage of 256× and 50×, respectively. The final assembled genome is about 583.47 Mb with contig N50 sizes of 2.87 Mb, which accounts for 91.44% of the estimated genome size of 638.07 Mb. Using Hi-C–based chromatin contact maps, 99.31% of the genome assembly was placed into 25 chromosomes, and the N50 is 22.3 Mb. The gene annotation completeness was evaluated by BUSCO, and 2,470 of the 2,586 conserved genes (95.5%) could be found in our assembly. Repetitive elements were calculated to reach 33.08% of the whole genome. Moreover, we identified 25,406 protein-coding genes, of which 92.59% have been functionally annotated. This genome assembly will be a valuable genomic resource to understand the biology of the Tibetan loaches and will also set a stage for comparative analysis of the classification, diversification, and adaptation of fishes in Cobitoidea.

## Introduction

**Figure 1:**
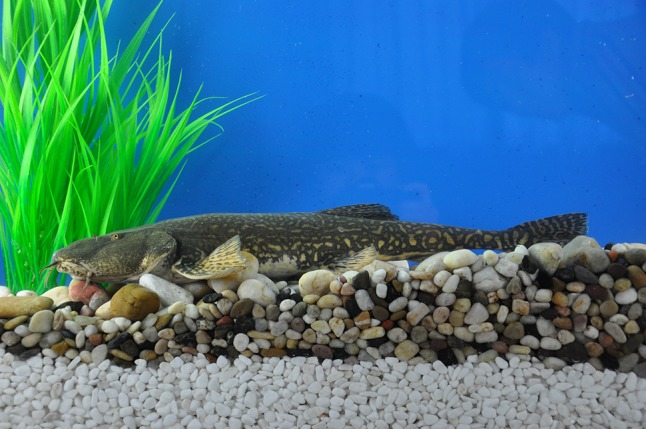
Photograph of the Tibetan loach, *Triplophysa siluroides*.

The fish superfamily Cobitoidea is one of the two superfamilies of the order Cypriniformes, which is the largest monophyletic group of freshwater fishes in the world (Nelson et al., 2016). The classification and relationship of Cobitoidea are still under debate based on morphological and few molecular markers, for example, which families constitute the Cobitoidea (Tang et al., 2006; Slechtova et al., 2007; Mayden et al., 2009; Kottelat, 2012; Nelson et al., 2016). Therefore, it is essential to investigate the relationship of Cobitoidea fishes at the genomic level. Compared with the many genome sequences released from fishes in Cyprinoidea, there are still few genomes that have been sequenced yet for Cobitoidea fishes, which hampers remarkably further comparative analyses of all Cobitoidea fishes. As ecologically and commercially important freshwater species, some fishes of the superfamily Cobitoidea play important roles in the commercial fisheries on China, such as oriental weatherloach (*Misgurnus anguillicaudatus*) (Chen et al., 2014) and giant stone loach (*Triplophysa siluroides*) (**Figure 1**) (Zhu, 1989; Chen et al., 2016).

The giant stone loach belongs to the family Nemacheilidae (Cypriniformes) and is an endemic species restricted to the upper reaches of the Yellow River drainage in China (Ding, 1994). It is the biggest member of balitorid loachs in the world attaining about 0.5 m in total length and weight of about 1.5 kg (Zhu, 1989) and was previously an important economic fish in its distribution regions (Zhu, 1989; Chen et al., 2016). However, the natural population of the giant stone loach has reduced sharply in recent years because of heavy fishing and habitat destruction, which makes it a vulnerable species in the China Red Data Book of Endangered Animals (Wang et al., 1998; Wang and Xie, 2004; He et al., 2008). Thus, it is in urgent need to perform genetic analysis on the giant stone loach to protect their natural resources, especially at the genomic level. However, so far, only transcriptome, mitochondrial genome, and SNP data have been reported for the giant stone loach (Wang et al., 2015; Chen et al., 2016; Chen et al., 2018). It is thus essential to sequence the genome of the giant stone loach, which will help protect this species, identify functional genes controlling important economic traits, reveal the genetic basis of adaptation to the extreme environments of the Tibetan Plateau, and, most importantly, provide a reference genome for the Cobitoidea fishes.

In this work, we integrated genomic sequencing data from Illumina short reads and PacBio long reads to generate a reference genome for *T*. *siluroides*. The completeness and continuity of this chromosome level genome were comparable with other model fish species, which will definitely provide valuable genomic resources for studies for the evolution and adaptation of Cobitoidea fishes.

## Materials and Methods

### Tissue Sampling and Ethics Statement

Tissue for genome sequencing of *T*. *siluroides* (NCBI taxonomy ID: 422203) was sampled from a single individual collected from the Yellow River at Gansu Province in China (33°25′N, 102°17′E). Muscle was collected and frozen in liquid nitrogen. All animal experimental procedures were approved by the ethics committee of Institute of Hydrobiology, Chinese Academy of Sciences.

### Library Construction and Sequencing

Genomic DNA was extracted from the muscle tissue using Qiagen GenomicTip100 (Qiagen, Hilden, Germany). For Illumina sequencing, we constructed a total of seven libraries with four short-insert libraries (170, 220, 320, and 600 bp) and three long-insert libraries (2, 5, and 10 kb) using the standard protocol provided by Illumina (San Diego, CA, USA). Paired-end sequencing was performed using the Illumina HiSeq 2000 platform for each library.

For the long insert size library, we sequenced it on a PacBio Sequel instrument with Sequel SMRT cells 1M v2 (Pacific Biosciences, Menlo Park, CA, USA) with one movie of 600 min at the Genome Center of Nextomics (Wuhan, China). In brief, approximately 5 µg of DNA was used to construct one single-molecule real-time (SMRT) library with an insert size of 20 kb. The library was sequenced in five SMRT DNA sequencing cells.

### Genome Size Estimation and Genome Assembly

We estimated the genome size based on the 17-mer depth frequency distribution method (Liu et al., 2013) with the following formula: genome size = k-mer_number/k-mer_depth (k-mer_number is the total number of k-mer from the sequencing data, and k-mer_depth is the peak frequency that was higher than any other frequencies).

Hybrid assembly of Illumina short reads and PacBio Sequel long reads was performed using the programs Platanus (Kajitani et al., 2014) and DBG2OLC (Ye et al., 2016). In short, the high-quality paired-end reads were used to construct accurate de Bruijin graph contigs using the program Platanus (Kajitani et al., 2014). Then, the program DBG2OLC (Ye et al., 2016) was used to map short contigs to PacBio Sequel long reads and generate a hybrid assembly. We further corrected the mixed assembly results by Pilon (Walker et al., 2014), with default parameters. Finally, the program SSPACE (Boetzer et al., 2011) was used to scaffold the hybrid assembly by incorporating mate pair reads.

### Genome Scaffolding With Chromatin Contact Maps

The processes of crosslinking, lysis, chromatin digestion, biotin marking, proximity ligations, crosslinking reversal, and DNA purification steps were used in previous studies (Dudchenko et al., 2017). Briefly, the fresh fish muscle sample was treated with 1% formaldehyde for 10 min at room temperature to perform cross-linking. The reaction was then quenched by adding 2.5 M glycine to 0.2 M for about 5 min. Nuclei were further digested with 100 units of DpnII and marked with biotin-14-dCTP (Invitrogen) and then ligated by T4 DNA ligase. After incubating overnight to reverse cross-links, the ligated DNA was then sheared to 300- to 600-bp fragments. The DNA fragments were further blunt-end repaired and A-tailed, followed by purification through biotin-streptavidin–mediated pull-down. Finally, the Hi-C libraries were quantified and sequenced on the Illumina HiSeq X Ten platform (San Diego, CA, USA) with 150 paired-end mode. The sequencing reads were mapped to the hybrid genome assembly with BWA (Li and Durbin, 2010), and uniquely mapped read pairs were retained. Contigs from the hybrid genome assembly were clustered, ordered, and oriented using Proximo (Burton et al., 2013).

### Assessment of Genome Completeness

The completeness of our *de novo* genome assembly was evaluated using benchmarking universal single-copy orthologs (BUSCO, v3) (Simao et al., 2015), which quantitatively assesses genome completeness using evolutionarily highly conserved 2,586 single-copy vertebrate genes. We also assessed the percentage of reads covered in our genome assembly by mapping the high-quality Illumina reads for short insert size libraries onto the *de novo* genome assembly using bwa (Li and Durbin, 2010) with default parameters.

### Repeat Annotation

We analyzed the repetitive sequences in *T*. *siluroides* genome with a combination of *de novo* and homology-based methods. First, we constructed a *de novo* repeat library using the RepeatModeller (v. 1.05) (Tarailo-Graovac and Chen, 2009) and LTR FINDER (Xu and Wang, 2007) with default parameters. Then, we mapped our assembled genome sequences against the constructed *de novo* repeat libraries and the RepBase (v. 21.01) (Jurka et al., 2005) to detect the novel and known transposable elements using the RepeatMasker (v. 4.06) (Tarailo-Graovac and Chen, 2009). Meantime, we employed the Tandem Repeat Finder (v. 4.04) (Benson, 1999) to predict the tandem repeats. Finally, we used the RepeatProteinMask software (v. 4.0.6) (Tarailo-Graovac and Chen, 2009) to annotate transposable element relevant proteins in our genome assembly.

### Gene Annotation

To annotate the structures and functions of putative genes in *T*. *siluroides* genome assembly, we used both *ab initio* prediction and homology-based prediction methods. For *ab initio* prediction, we used Augustus (Stanke et al., 2006), GenScan (Burge and Karlin, 1997), and glimmerHMM (Majoros et al., 2004) programs to analyze the repeat-masked *T*. *siluroides* genome assembly. For homology-based prediction, homologous protein sequences of cave fish (*Astyanax mexicanus*) (McGaugh et al., 2014), zebrafish (*Danio rerio*, GRCz10) (Howe et al., 2013), medaka (*Oryzias latipes*) (Kasahara et al., 2007), and Japanese puffer (*Fugu rubripes*) (Aparicio et al., 2002) were obtained from Ensembl (release 89) (Cunningham et al., 2015) and aligned to the repeat-masked *T*. *siluroides* genome using TblastN (version 2.2.26) with an *E* value cutoff of 1e−5. Then, the aligned sequences and corresponding query protein were filtered and passed to Genewise (version 2.4.1) (Birney et al., 2004) to predict the potential gene structures on all alignments. Finally, the above two gene sets were integrated to yield a comprehensive and nonredundant gene set using EVidenceModeler (EVM, version 1.1.1) (Haas et al., 2008).

Then, gene functional annotations were performed by aligning translated gene coding sequences to known databases, including SwissProt and TrEMBL, Gene Ontology (GO), InterProScan, and Kyoto Encyclopedia of Genes and Genomes (KEGG), using BLASTP (version 2.2.26) with an *E* value of 1e−5.

In addition, we also identified noncoding RNA genes in the *T*. *siluroides* genome. We used blast to search rRNA against the rRNA database and tRNAscan-SE (Lowe and Eddy, 1997) to search tRNA in the genome sequences. We also used blast to search miRNA and snRNA genes *via* the Rfam database (Gardner et al., 2011).

### Phylogenetic Analysis

Protein sequences of 11 ray-finned fishes (D. rerio, Gasterosteus aculeatus, Lepisosteus oculatus, Oreochromis niloticus, O. latipes, Takifugu rubripes, Xiphophorus maculatus, A. mexicanus, Gadus morhua, Poecilia formosa, and Tetraodon nigroviridis) were downloaded from the Ensembl database (Release 90), and the protein sequences of Hippocampus comes (Lin et al., 2016) and Boleophthalmus pectinirostris (You et al., 2014) were acquired from the authors. The longest coding sequence was chosen to represent each gene. We first performed all-against-all comparison of all proteins using BLASTP (version 2.2.26) with a cutoff of E value <1e−5 to both genes and then clustered the genes into gene families using solar and hcluster_sg in TreeFam (Li et al., 2006). Subsequently, we extracted the one-to-one orthologous genes from the aforementioned 14 species. The protein sequences of these orthologous genes were aligned using MUSCLE (Edgar, 2004) with the default parameters. We then converted the protein alignments to their corresponding coding sequences (CDSs) using an in-house perl script. All these aligned nucleotide sequences were then concatenated into a supergene. Next, the 4D sites (fourfold degenerate sites) were extracted from the supergenes to construct a phylogenetic tree using RAxML (Stamatakis, 2014) with the GTR+G+I model.

## Results and Discussion

In total, we generated about 252.90 Gb of raw Illumina reads, including 43.96, 40.33, 43.64, 42.74, 27.84, 27.81, and 26.58 Gb of reads from the 170-, 220-, 320-, 600-, 2k-, 5k-, and 10-kb libraries, respectively (**Table S1**). We also generated about 31.60 Gb of raw PacBio long data with an average read length of 10,563 bp (**Table S2**). After removal of low-quality and redundant reads, 163.37 Gb of clean Illumina reads and 31.26 Gb of clean PacBio reads were obtained for genome assembly (**Tables S1 and S2**). The genome size estimated by k-mer analysis was approximately 638 Mb, with the main peak at a depth of 183× (**Figure S1 and Table S3**). The small peak at a depth of 92 indicated that the genome heterozygosity of *T. siluroides* was low (0.29%).

We assembled the genome with the hybrid method of Illumina short reads and PacBio Sequel long reads using the programs Platanus (Kajitani et al., 2014) and DBG2OLC (Ye et al., 2016). The final *de novo* assembly for the *T*. *siluroides* has a total length of 583.47 Mb, representing 91.44% of the estimated genome size, with contig N50 length of 2.87 Mb and the longest contig length 14.65 Mb (**Table 1**), which makes it one of the most high-quality genome assemblies currently available.

**Table 1:** Summary of genome assembly of *T. siluroides*.

Terms	Size (bp)	Number
N90	452,639	251
N80	881,594	163
N70	1,446,248	112
N60	2,005,727	77
N50	2,872,994	53
Max length	14,649,642	—
Total length	583,471,586	—
Total number	—	1,004
Total number (≥1 kb)	—	1,004
Total number (≥5 kb)		1,001

**Figure 2:**
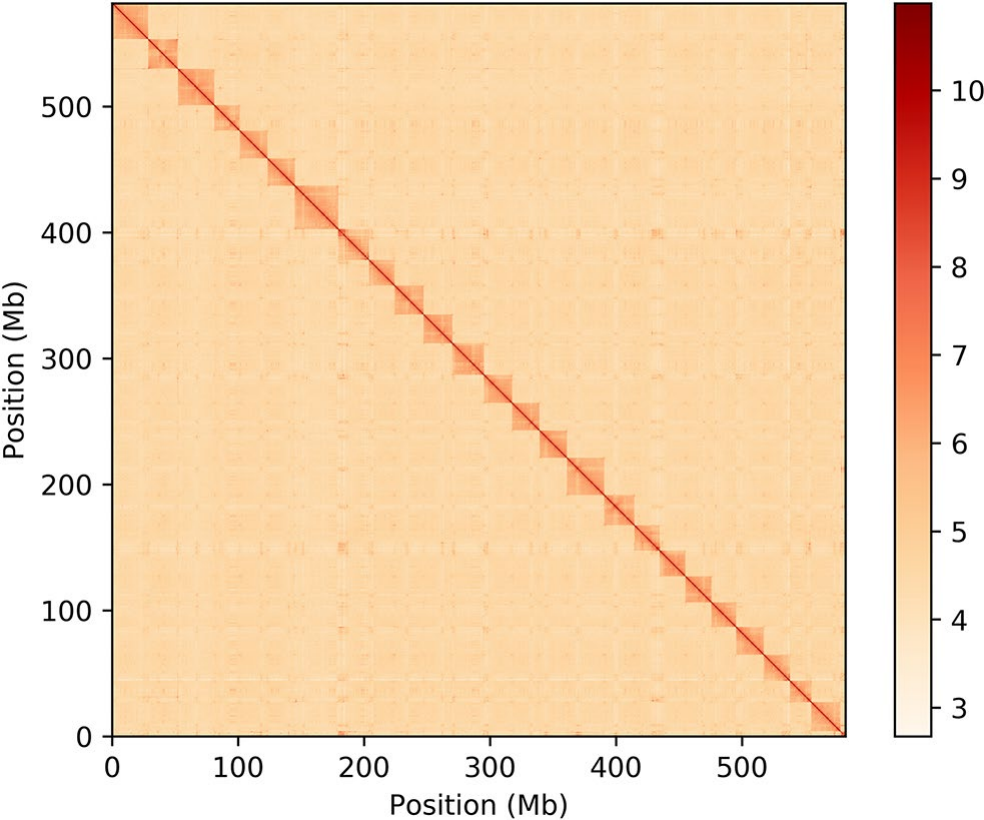
Hi-C interaction heat map showing the clustering of the primary hybrid contigs into 25 sets of chromosome-scale superscaffolds.

To construct a chromosome-scale reference genome assembly of the Tibetan loach, chromatin contact maps were produced by Frasergen Information Co. Ltd. (Wuhan, China) (**Figure 2**). We sequenced a total of 65.39 Gb of HiSeq data and obtained 27.7 Gb valid data (43.95%) that could be used to anchor the contigs into chromosomes. The contig clustering allowed the placement of 856 contigs into 25 scaffolds (chromosomes) with lengths ranging from 16.67 to 34.49 Mb (**Table S4**). While only 81.99% of the contigs were anchored to chromosomes, this corresponds to 99.31% of the total length of primary hybrid genome assembly. This genome scaffolding step improved substantially the primary assembly contiguity, raising the N50 approximately 8.3-fold from 2.7 Mb to 22.3 Mb (**Table 2**).

**Table 2:** Chromosome metrics before and after Hi-C scaffolding.

Terms	Contig (original)	Scaffold (Hi-C)
Number	1,004	856
N50	2,872,994	22,312,937
Total length	582,350,959	578,738,912

The completeness of our *de novo* genome assembly was assessed by BUSCO, which showed that 98.4% of the 2,586 highly conserved single-copy genes can be detected in the *T*. *siluroides* genome, with 95.5% and 2.9% identified as complete and fragmented, respectively (**Table S5**). We also found that 98.36% of the high-quality Illumina reads can be mapped onto the *de novo* genome assembly (**Table S6**). These results suggested that the quality of our *de novo* assembled genome was high for both completeness and base level accuracy.

A total of 193 Mb of nonredundant repetitive sequences are identified in *T*. *siluroides* genome, which account for 33.08% of the whole genome. The percentage of repetitive sequences is similar to other fish species (Chalopin et al., 2015). The most predominant repeat is the DNA transposons, which account for 12.58% (73.38 Mb in total) of the genome (**Table S7, Table S8, Figure S2, and Figure S3**). The fraction of DNA transposons in *T*. *siluroides* genome is in good agreement with those in other fish species, which indicated that the fraction of DNA transposons in fish genomes (10%) is significantly higher than those in mammals (3%) (Chalopin et al., 2015).

After the characterization of repetitive sequences in the *T*. *siluroides* genome assembly, gene annotation was performed by using both *ab initio* prediction and homology-based prediction methods. In total, 25,406 protein-coding genes were identified (**Table S9, Figure S4, and Figure S5**). Approximately 92.59% of the predicted genes were successfully annotated using five protein databases: InterPro (83.40%), GO (67.67%), KEGG (69.67%), Swiss-Prot (85.84%), and TrEMBL (92.38%) (**Table S10**). Finally, we identified noncoding RNA genes in the *T*. *siluroides* genome and found that a total of 6,822 microRNAs (miRNA), 6,513 transfer RNA (tRNA), 8,053 ribosomal RNA (rRNA), and 12,655 snRNA genes could be detected in the *T*. *siluroides* genome (**Table S11**).

**Figure 3:**
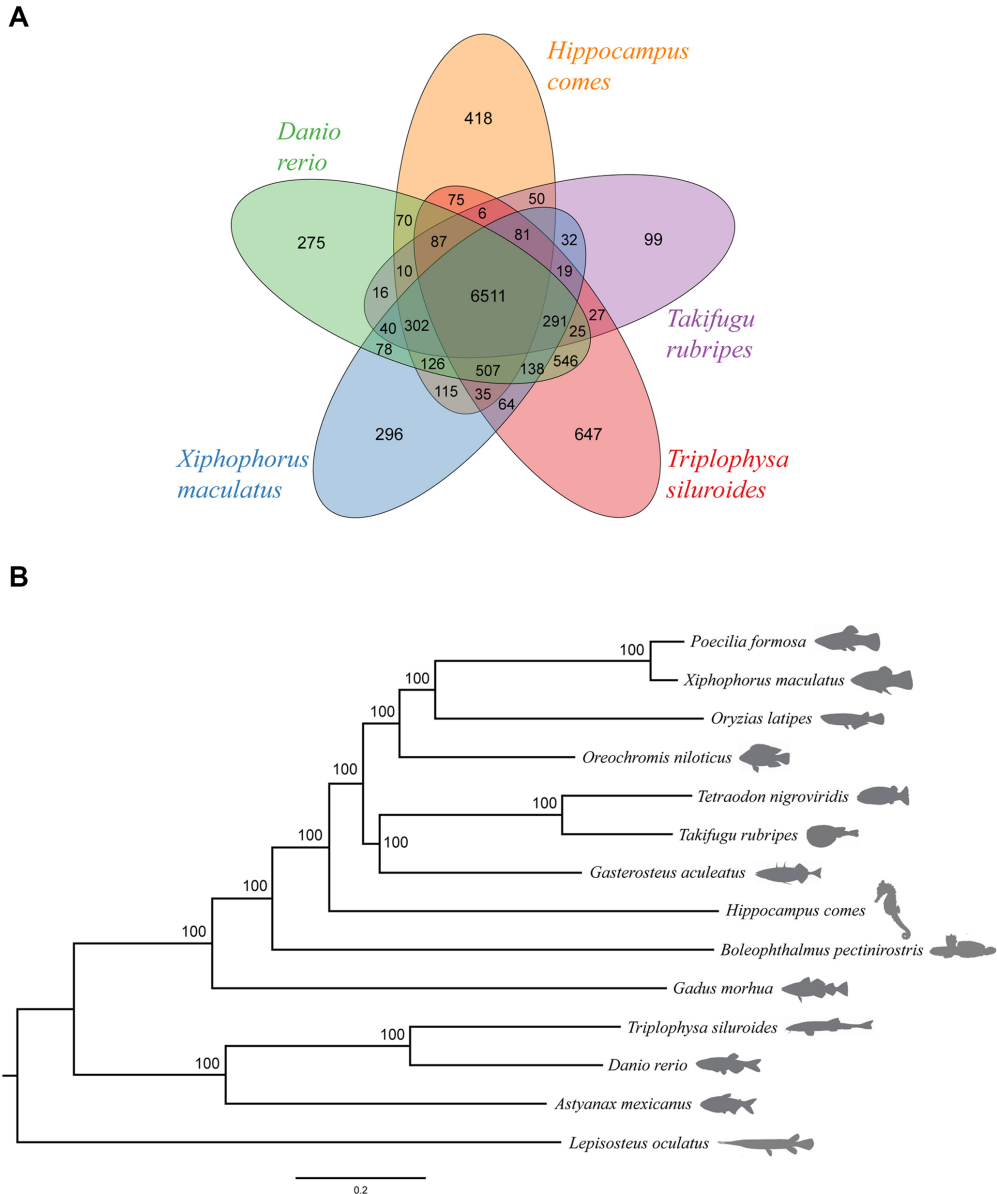
Genome evolution. **(A)** A Venn diagram of the orthologous gene families shared among five ray-finned fish genomes (*Danio rerio*, *Hippocampus comes*, *Takifugu rubripes*, *Triplophysa siluroides*, *Xiphophorus maculatus*). **(B)** Phylogeny of the 14 ray-finned fishes with the spotted gar as the outgroup. The bootstrap support value for the topology is 100.

We further obtained the gene families for 14 fish species and then classified these gene families for a subset of five species (*D. rerio*, *H. comes*, *T. rubripes*, *X. maculatus*, and *T. siluroides*) (**Figure 3A**). In brief, the 25,406 protein-coding genes in *T. siluroides* comprised 2,104 single-copy orthologs, 14,222 multiple-copy orthologs, 1,003 unique paralogs, 6,122 other orthologs, and 1,955 unclustered genes (**Figure S6**). Furthermore, 9,225 gene families were identified in the *T. siluroides* genome, and 300 of these were found to be unique in *T. siluroides* genome (**Table S12**). We found that the *T. siluroides* species-specific gene families were mainly enriched in the following GO categories, including immune response, energy metabolism, and hormone activity, implying that species-specific genes may play important roles in *T. siluroides* adaptation to the extreme environments on the Tibetan Plateau. Based on the TreeFam gene clusters and MUSCLE multiple alignment, 1,087 one-to-one orthologs were identified from the 14 fish genomes. Phylogenetic analysis from these orthologs supported the placement of *T. siluroides* close to zebrafish (**Figure 3B**).

## Conclusion

We report the high-quality whole genome sequencing, assembly, and annotation of the Tibetan loach (*T. siluroides*). The high-quality genome assembly will provide a valuable resource for studying the genetic mechanisms of adaptation to the Tibetan Plateau in fishes.

## Data Availability Statement

The raw sequencing reads of all libraries have been deposited in the SRA database (SRP198880).

## Ethics Statement

The animal study was reviewed and approved by The ethics committee of Institute of Hydrobiology, Chinese Academy of Sciences.

## Author Contributions

SH and LY designed the study. YZ coordinated the study. YW and TW collected the sample. SD and YD performed the bioinformatics analysis. LY and YW analyzed the results and wrote the manuscript with inputs from the other authors. All authors read and approved the final manuscript.

## Conflict of Interest

Authors SD and YD were employed by Nowbio Biotechnology Company, Kunming, Yunnan, China.

The remaining authors declare that the research was conducted in the absence of any commercial or financial relationships that could be construed as a potential conflict of interest.

The reviewer QQ declared a past co-authorship with two of the authors YD, SH to the handling editor.
