# Supplementary material for: A Chromosome-Scale Reference Assembly of a Tibetan Loach, Triplophysa siluroides
Source: Front Genet. 2019 Oct 16;10:991. doi: 10.3389/fgene.2019.00991 (PMC6807559; doi:10.3389/fgene.2019.00991)
Supplement: Supplementary file 1 [file DataSheet_1.docx]

**Supporting information**


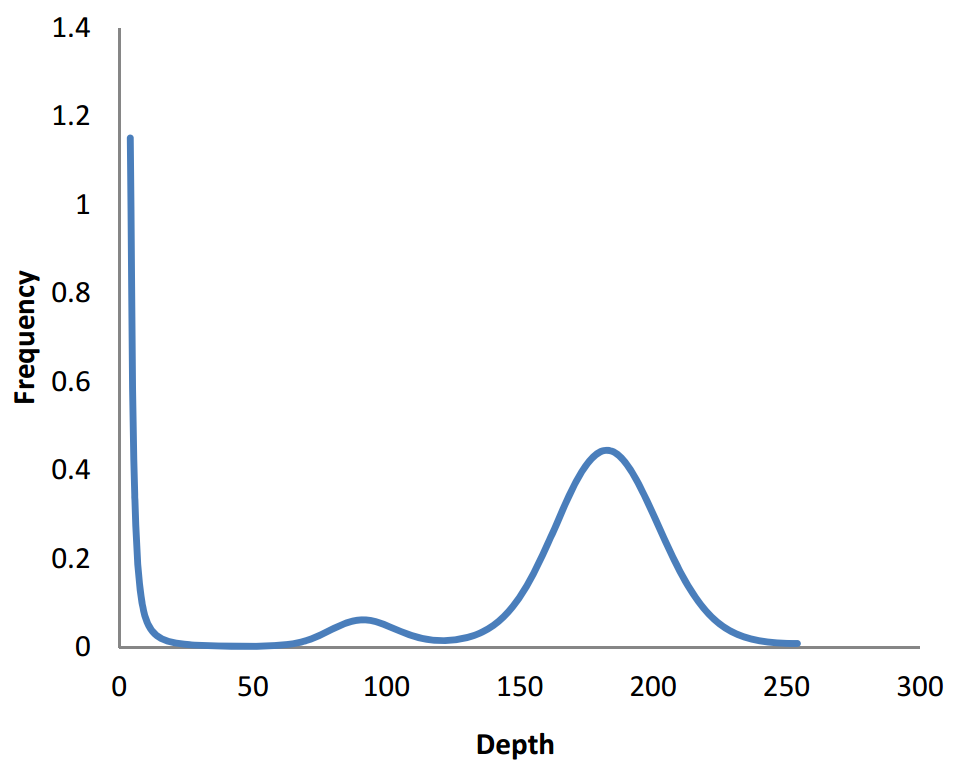


**Figure S1 K-mer (k=17) distribution in *Triplophysa siluroides*.** The x-axis is depth (X); the y-axis is the proportion which represents the frequency at that depth divide by the total frequency of all the depth.

**
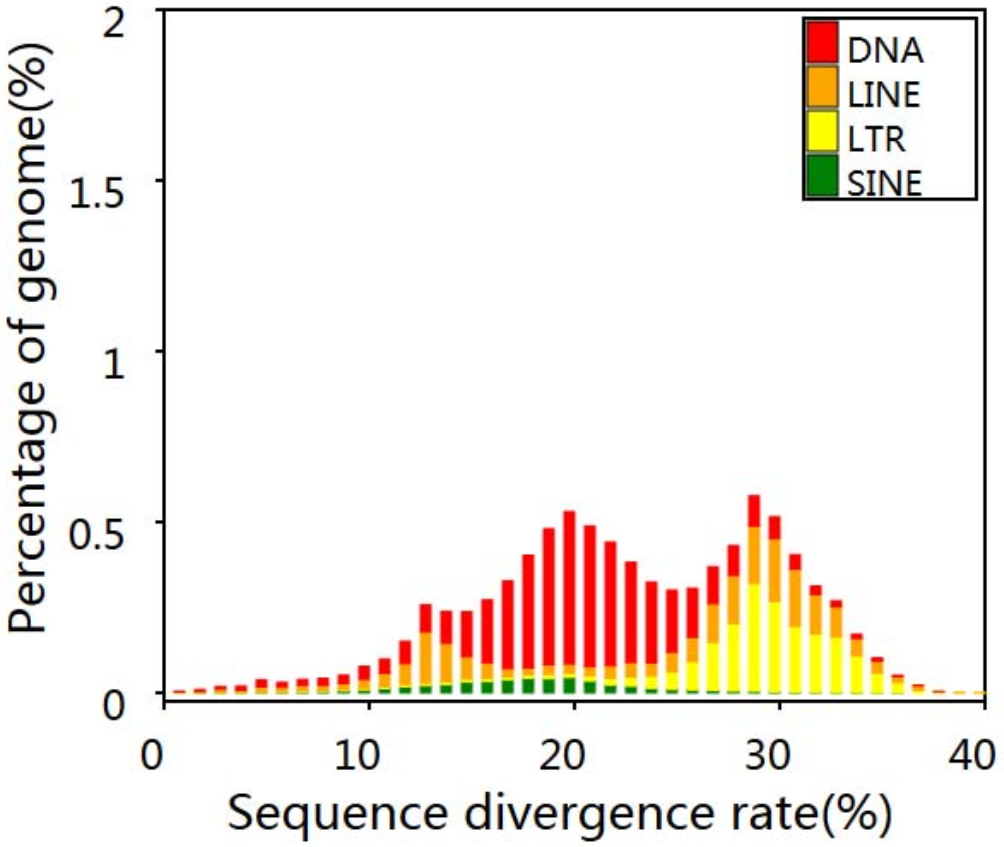
**

**Figure S2 Distribution of Divergence Rate of each Type of *T.* *siluroides*’s TE (*De novo*).** The divergence rate was calculated between the identified TE elements in the genome by homology-based method and the consensus sequence in the Repbase.

**
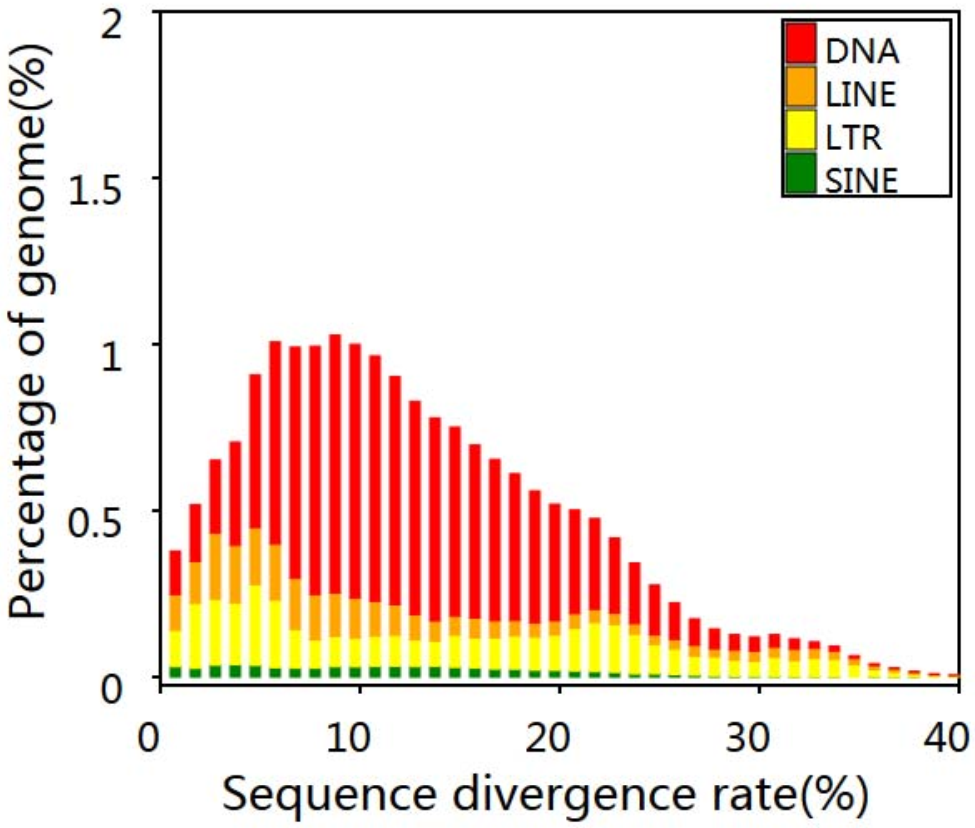
**

**Figure S3 Distribution of Divergence Rate of each Type of *T.* *siluroides*’s TE.** The divergence rate was calculated between the identified TE elements in the genome by *de novo* method and the consensus sequence in the predicted TE library.


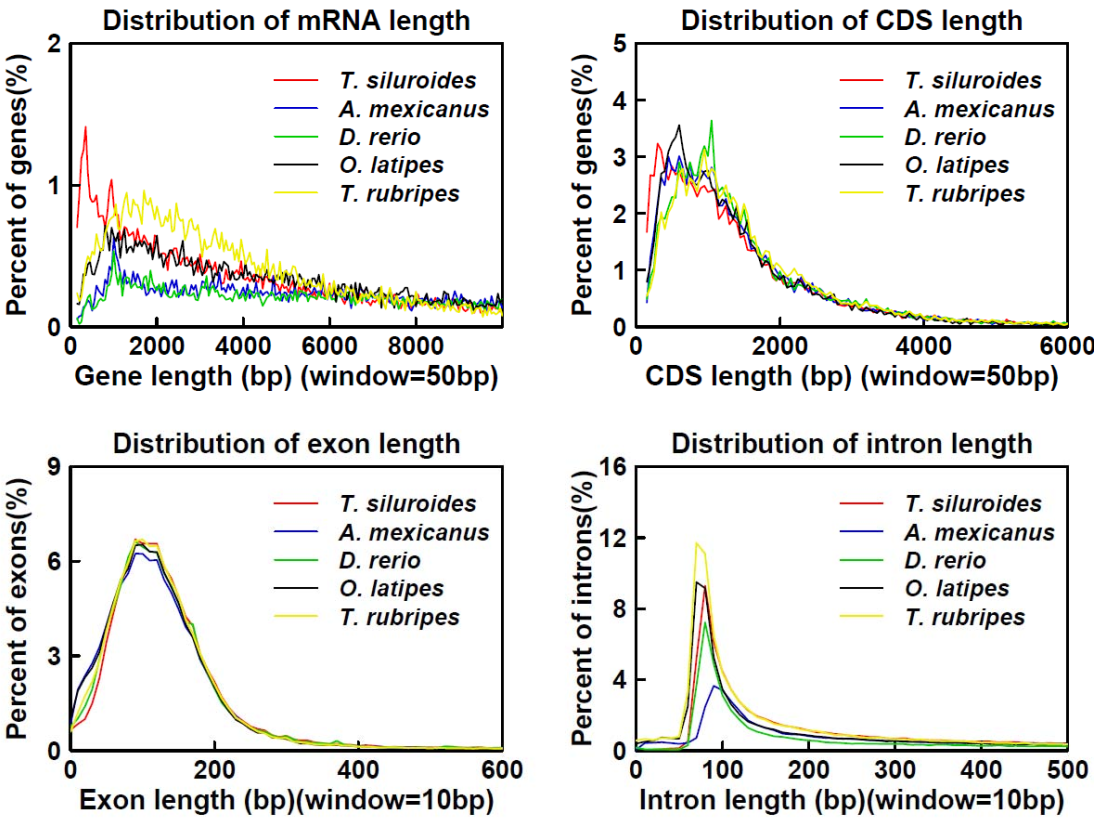


**Figure S4 The comparisons of mRNA length, CDS length, exon length, and intron length for protein-coding genes in genomes of *T.* *siluroides* and other teleosts.**

**
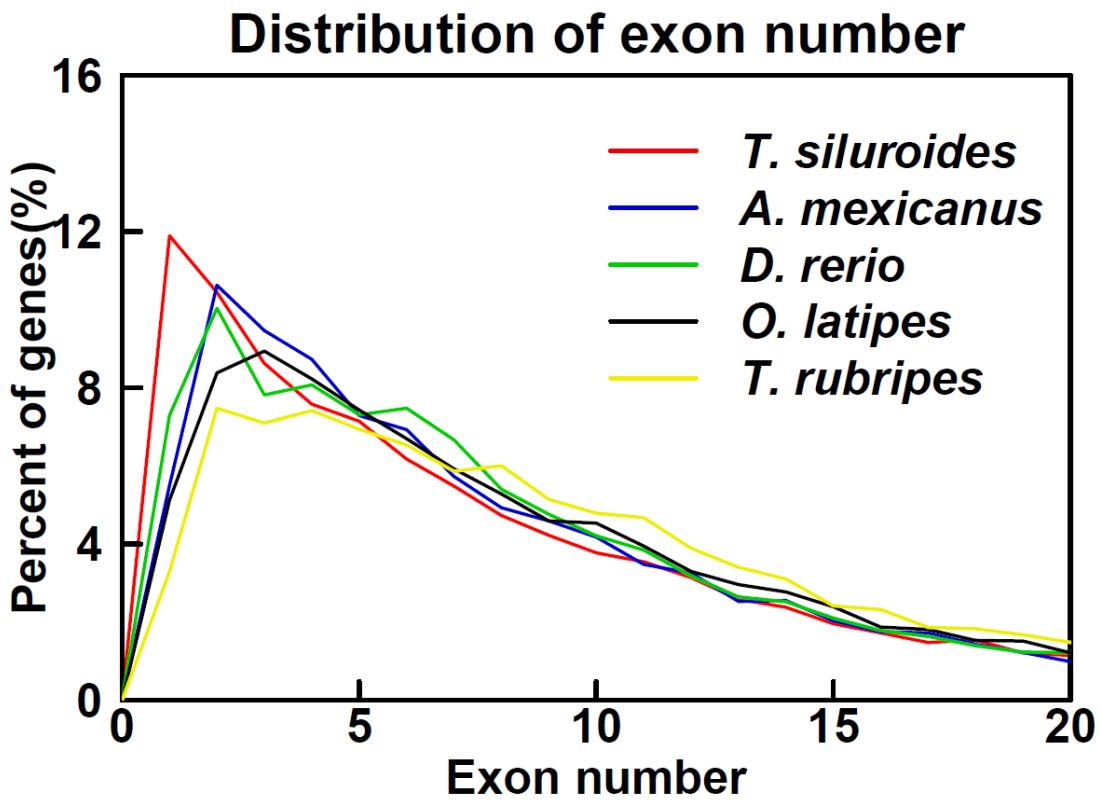
**

**Figure S5 The comparisons of exon number for protein-coding genes in genomes of *T.* *siluroides* and other teleosts.**

**
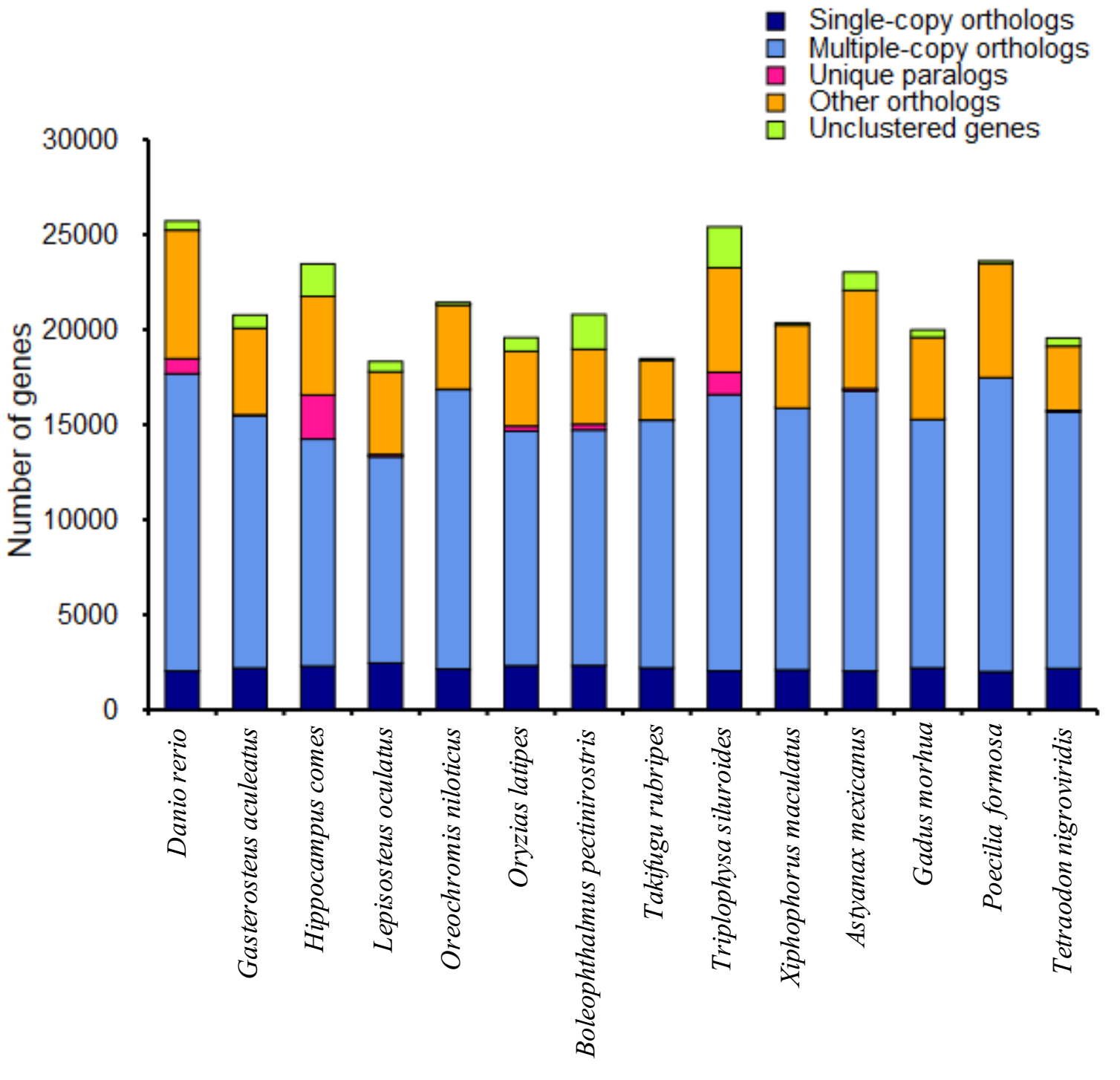
Figure S6 Gene number distribution for gene family clustering.** The color scheme represented various categories of gene families, and the dark blue referred to the single-copy gene families.

**Table S1 Summary of Illumina platform reads used in the sequencing of the *T.* *siluroides* genome.**

|  |  | **Raw Reads** | | **Qualified Reads** | |
| --- | --- | --- | --- | --- | --- |
| **Insert Size (bp)** | **Reads Length (bp)** | **Bases sequenced (Gb)** | **Sequence coverage (×)** | **Bases sequenced (Gb)** | **Sequence coverage (×)** |
| 170 | 100 | 43.96 | 68.90 | 37.39 | 58.61 |
| 220 | 100 | 40.33 | 63.21 | 34.96 | 54.80 |
| 320 | 100 | 43.64 | 68.40 | 36.34 | 56.96 |
| 600 | 100 | 42.74 | 66.99 | 31.73 | 49.73 |
| 2,000 | 90 | 27.84 | 43.64 | 7.50 | 11.76 |
| 5,000 | 90 | 27.81 | 43.59 | 8.46 | 13.26 |
| 10,000 | 90 | 26.58 | 41.66 | 6.99 | 10.96 |
| Total |  | 252.90 | 396.39 | 163.37 | 256.07 |

**Table S2 Summary of PacBio Sequel platform reads used in the sequencing of the *T.* *siluroides* genome.**

|  | **Subreads Bases (nt)** | **Subreads Num.** | **Avg. Subread length (bp)** | **Subreads N50** | **Sequence coverage (X)** |
| --- | --- | --- | --- | --- | --- |
| **Raw** | 31,602,394,527 | 2,971,157 | 10,563 | 19,350 | 49.53 |
| **Filtered** | 31,263,636,776 | 3,914,538 | 7,986 | 13,466 | 49.00 |

**Table S3 Statistics of 17-mer analysis for the *T.* *siluroides* genome.**

| **K-mer** | **K-mer num** | **Peak Depth** | **Genome Size** | **Used Bases** | **Used Reads** | **Coverage (X)** |
| --- | --- | --- | --- | --- | --- | --- |
| 17 | 116,766,698,288 | 183 | 638,069,389 | 1.40×10^11^ | 1.48×10^9^ | 220 |

**Table S4 Chromosome metrics after Hi-C scaffolding of the primary assembly.**

| **Chromosome** | **Number of scaffold** | **Length of scaffold** | **Length of chromosome** |
| --- | --- | --- | --- |
| **chr1** | 70 | 28,545,376 | 28,579,876 |
| **chr2** | 28 | 23,611,745 | 23,625,245 |
| **chr3** | 35 | 29,105,257 | 29,122,257 |
| **chr4** | 22 | 20,301,957 | 20,312,457 |
| **chr5** | 29 | 22,008,727 | 22,022,727 |
| **chr6** | 22 | 21,638,684 | 21,649,184 |
| **chr7** | 47 | 34,488,196 | 34,511,196 |
| **chr8** | 145 | 24,248,481 | 24,320,481 |
| **chr9** | 27 | 20,314,950 | 20,327,950 |
| **chr10** | 16 | 23,282,402 | 23,289,902 |
| **chr11** | 23 | 22,709,626 | 22,720,626 |
| **chr12** | 36 | 25,097,937 | 25,115,437 |
| **chr13** | 40 | 22,293,437 | 22,312,937 |
| **chr14** | 29 | 21,894,357 | 21,908,357 |
| **chr15** | 18 | 21,848,559 | 21,857,059 |
| **chr16** | 21 | 29,407,776 | 29,417,776 |
| **chr17** | 13 | 24,109,232 | 24,115,232 |
| **chr18** | 70 | 20,566,348 | 20,600,848 |
| **chr19** | 25 | 20,309,634 | 20,321,634 |
| **chr20** | 16 | 20,749,552 | 20,757,052 |
| **chr21** | 17 | 19,327,181 | 19,335,181 |
| **chr22** | 19 | 22,201,744 | 22,210,744 |
| **chr23** | 49 | 20,686,867 | 20,710,867 |
| **chr24** | 27 | 16,670,781 | 16,683,781 |
| **chr25** | 12 | 22,904,606 | 22,910,106 |
| **Total** | 856 | 578,323,412 | 578,738,912 |

**Table S5 BUSCO scores of the assembled *T.* *siluroides* genome when using the BUSCO v3 vertebrate dataset.**

|  | **Count** | **Ratio** |
| --- | --- | --- |
| **Complete BUSCOs** | 2470 | 95.5% |
| **Complete and single-copy BUSCOs** | 2395 | 92.6% |
| **Complete and duplicated BUSCOs** | 75 | 2.9% |
| **Fragmented BUSCOs** | 75 | 2.9% |
| **Missing BUSCOs** | 41 | 1.6 % |
| **Total BUSCO groups searched** | 2586 | - |

**Table S6 Statistic of reads aligned onto *Triplophysa siluroides* genome assembly.**

| **Insert Size (bp)** | **Total Reads** | **Mapped Reads** | **Mapped Ratio(%)** |
| --- | --- | --- | --- |
| **170** | 392,765,546 | 385,744,038 | 98.21 |
| **220** | 367,254,506 | 361,527,425 | 98.44 |
| **320** | 380,930,904 | 374,960,541 | 98.43 |
| **600** | 332,157,932 | 326,687,697 | 98.35 |
| **Total** | 1,473,108,888 | 1,448,919,701 | 98.36 |

**Table S7 Statistics of repeat content in *T.* *siluroides* genome.**

| **Type** | **Repeat size (bp)** | **% of genome** |
| --- | --- | --- |
| **Trf** | 12,975,698 | 2.22 |
| **Repeatmasker** | 49,909,217 | 8.55 |
| **Proteinmask** | 37,959,681 | 6.51 |
| ***De novo*** | 173,073,985 | 29.66 |
| **Total** | 192,996,777 | 33.08 |

**Table S8 Summary statistics of repetitive sequences in *T.* *siluroides* genome.**

|  | **Repbase TEs** | | **TE proteins** | | ***De novo*** | | **Combined TEs** | |
| --- | --- | --- | --- | --- | --- | --- | --- | --- |
| **Type** | **Length**  **(bp)** | **% in genome** | **Length**  **(bp)** | **% in genome** | **Length**  **(bp)** | **% in genome** | **Length**  **(bp)** | **% in genome** |
| **DNA** | 24,390,065 | 4.18 | 6,813,148 | 1.17 | 68,047,834 | 11.66 | 73,382,441 | 12.58 |
| **LINE** | 11,919,222 | 2.04 | 14,517,557 | 2.49 | 16,188,972 | 2.77 | 22,791,032 | 3.91 |
| **SINE** | 2,719,663 | 0.47 | 0 | 0.00 | 4,136,631 | 0.71 | 4,670,579 | 0.80 |
| **LTR** | 11,539,814 | 1.98 | 16,724,586 | 2.87 | 21,296,146 | 3.65 | 27,011,221 | 4.63 |
| **Other** | 497 | 0.00 | 0 | 0.00 | 0 | 0.00 | 497 | 0.00 |
| **Unknown** | 0 | 0.00 | 0 | 0.00 | 64,960,817 | 11.13 | 64,960,817 | 11.13 |
| **Total** | **49,909,217** | **8.55** | **37,959,681** | **6.51** | **171,322,425** | **29.36** | **184,694,239** | **31.65** |

**Table S9 General statistics of predicted protein-coding genes.**

| **Methods** | **Gene Number** | **Avg. mRNA Length** | **Total Exon Number** | **Avg. Exon Length** | **Avg. CDS Length** | **Avg. Exon Number** | **Total Intron Length** |
| --- | --- | --- | --- | --- | --- | --- | --- |
| ***ab initio*** |  |  |  |  |  |  |  |
| augustus | 13,184 | 7966.90 | 84,205 | 148.41 | 947.90 | 6.39 | 92,538,466 |
| genscan | 31,266 | 13076.71 | 261,050 | 186.12 | 1553.97 | 8.35 | 360,270,080 |
| glimmerHMM | 69,440 | 6116.13 | 326,341 | 153.36 | 720.74 | 4.70 | 374,655,896 |
| **Homology** |  |  |  |  |  |  |  |
| *A. mexicanus* | 22,346 | 9192.49 | 200,971 | 168.26 | 1513.27 | 8.99 | 171,599,949 |
| *D. rerio* | 23,281 | 9869.16 | 218,457 | 166.81 | 1565.22 | 9.38 | 193,324,175 |
| *O. latipes* | 20,907 | 8669.17 | 182,649 | 162.47 | 1419.35 | 8.74 | 151,571,922 |
| *T. rubripes* | 19,852 | 9705.39 | 186,914 | 161.98 | 1525.13 | 9.42 | 162,394,552 |
| **EVM** | **25,406** | **10311.35** | **227,633** | **171.52** | **1536.79** | **8.96** | **222,926,490** |

**Table S10 Summary statistics of gene function annotation.**

|  | | **number** | **percent (%)** |
| --- | --- | --- | --- |
| **Annotated** | **InterPro** | 21,189 | 83.40 |
|  | **GO** | 17,191 | 67.67 |
|  | **KEGG** | 17,700 | 69.67 |
|  | **Swissprot** | 21,808 | 85.84 |
|  | **TrEMBL** | 23,469 | 92.38 |
| **Number of annotated genes** | | 23,523 | 92.59 |
| **Number of unannotated genes** | | 1,883 | 7.41 |

**Table S11 Summary statistics of non-coding RNA annotation.**

| **Type** | | **Copy number** | **Average length(bp)** | **Total length(bp)** | **% of genome** |
| --- | --- | --- | --- | --- | --- |
| **miRNA** |  | 235 | 82.42 | 19,369 | 0.0033 |
| **tRNA** |  | 640 | 81.20 | 51,966 | 0.0089 |
| **rRNA** | rRNA | 84 | 98.31 | 8,258 | 0.0014 |
|  | 18S | 43 | 118.86 | 5,111 | 0.00088 |
|  | 28S | 41 | 76.76 | 3,147 | 0.00054 |
|  | 5.8S | 288 | 144.45 | 41,601 | 0.0071 |
|  | 5S | 182 | 146.57 | 26,675 | 0.0046 |
| **snRNA** | snRNA | 52 | 150.35 | 7,818 | 0.0013 |
|  | CD-box | 44 | 136.95 | 6,026 | 0.0010 |
|  | HACA-box | 235 | 82.42 | 19,369 | 0.0033 |
|  | Splicing | 640 | 81.20 | 51,966 | 0.0089 |

**Table S12 Statistics of gene families generated by TreeFam.**

| **Species** | **Genes number** | **Genes in families** | **Unclustered genes** | **Family number** | **Unique families** | | **Average genes per family** |
| --- | --- | --- | --- | --- | --- | --- | --- |
| *Danio rerio* | 25713 | 25232 | 481 | 9188 | 78 | 2.75 | |
| *Gasterosteus aculeatus* | 20769 | 20075 | 694 | 8625 | 16 | 2.33 | |
| *Hippocampus comes* | 23458 | 21749 | 1709 | 8625 | 119 | 2.52 | |
| *Lepisosteus oculatus* | 18326 | 17781 | 545 | 8590 | 36 | 2.07 | |
| *Oreochromis niloticus* | 21431 | 21284 | 147 | 8100 | 11 | 2.63 | |
| *Oryzias latipes* | 19580 | 18859 | 721 | 8167 | 64 | 2.31 | |
| *Periophthalmus modestus* | 20798 | 18965 | 1833 | 8365 | 106 | 2.27 | |
| *Takifugu rubripes* | 18472 | 18379 | 93 | 7662 | 4 | 2.40 | |
| *Xiphophorus maculatus* | 20356 | 20233 | 123 | 8701 | 2 | 2.33 | |
| *Astyanax mexicanus* | 23024 | 22065 | 959 | 9037 | 59 | 2.44 | |
| *Gadus morhua* | 19987 | 19589 | 398 | 8486 | 5 | 2.31 | |
| *Poecilia formosa* | 23607 | 23483 | 124 | 9087 | 7 | 2.58 | |
| *Tetraodon nigroviridis* | 19556 | 19129 | 427 | 7944 | 36 | 2.41 | |
| *Triplophysa siluroides* | 25406 | 23254 | 2152 | 9225 | 300 | 2.52 | |
